# Intrusive Memory Frequency and Related Inner Tension Following Dialectical Behavior Therapy or Cognitive Processing Therapy for Posttraumatic Stress Disorder: An e-Diary Study

**DOI:** 10.2196/81081

**Published:** 2025-12-08

**Authors:** Sara E Schmitz, Ulrich W Ebner-Priemer, Nikolaus Kleindienst, Franziska Friedmann, Martin Bohus, Regina Steil, Meike Müller-Engelmann, Matthias F Limberger, Lisa-Marie Hartnagel, Philip Santangelo, Kathlen Priebe

**Affiliations:** 1 Mental mHealth Lab, Institute of Sports and Sports Science, Karlsruhe Institute of Technology, Hertzstr. 16, Karlsruhe, 76187, Germany, 49 72160841976; 2 Department of Psychiatry and Psychotherapy, Central Institute of Mental Health, Heidelberg University, Mannheim, Germany; 3 Department of Psychosomatic Medicine and Psychotherapy, Central Institute of Mental Health, Heidelberg University, Mannheim, Germany; 4 German Center for Mental Health (DZPG), Partner Site Mannheim - Heidelberg - Ulm, Germany; 5 Department of Psychology, Humboldt-Universität zu Berlin, Berlin, Germany; 6 Institute of Psychology, Goethe University Frankfurt, Frankfurt, Germany; 7 Department of Psychology, Faculty of Human Sciences, Medical School Hamburg, Hamburg, Germany; 8 Department of Behavioural and Cognitive Sciences, University of Luxembourg, Esch-sur-Alzette, Luxembourg; 9 Department for Psychiatry and Neurosciences CCM, Charité - Universitätsmedizin Berlin, corporate member of Freie Universität Berlin and Humboldt-Universität zu Berlin, Berlin, Germany

**Keywords:** electronic diary, posttraumatic stress disorder, intrusive memories, dialectical behavior therapy, cognitive processing therapy

## Abstract

**Background:**

Intrusive memories are a core symptom of posttraumatic stress disorder (PTSD), yet their retrospective assessment is prone to biases, making real-time methods such as e-diaries essential. While trauma-focused treatments target intrusive symptoms, their efficacy has not yet been evaluated using real-time assessments.

**Objective:**

This study aimed to use e-diaries to assess and compare the effects of dialectical behavior therapy for PTSD (DBT-PTSD) and cognitive processing therapy (CPT) on intrusive memories and related inner tension in a large sample of women with childhood abuse–related PTSD and co-occurring borderline personality disorder (BPD) symptoms.

**Methods:**

In a multicenter randomized controlled trial, 193 women with PTSD related to childhood sexual or physical abuse and at least 3 BPD criteria were randomized to receive either DBT-PTSD or CPT. e-Diary assessments were conducted at 3 time points: before treatment, after 6 months, and after 12 months of therapy. At each time point, participants reported intrusive memories and related inner tension over 5 consecutive days using an event-based design.

**Results:**

Both intrusive memories and related inner tension decreased significantly over time (intrusions: ß=–0.53, *P*<.001; inner tension: ß=–0.15, *P*<.001). While reductions in intrusion frequency did not differ significantly between treatment groups (ß=0.05, *P*=.45), DBT-PTSD was associated with significantly greater reductions in intrusion-related inner tension compared with CPT (ß=–0.16, *P*<.001).

**Conclusions:**

This study provides the first real-time evaluation of trauma-focused PTSD treatments using e-diaries in daily life. Both interventions were associated with reduced intrusion frequency, while DBT-PTSD showed greater reductions in associated emotional distress—potentially reflecting its emphasis on emotion-regulation strategies and distress tolerance, which may be particularly relevant for individuals with comorbid BPD symptoms. These findings highlight the value of e-diaries for capturing treatment-related symptom change in ecologically valid contexts.

## Introduction

Intrusive memories, characterized by involuntary and distressing recollections of traumatic events, are a hallmark symptom of posttraumatic stress disorder (PTSD) [1]. These memories are often accompanied by intense emotional responses, dissociation, and heightened inner tension, which together exacerbate psychological distress [2]. Here, *inner tension* refers to a state of aversive inner arousal, agitation, or pressure that encompasses both psychological and physiological activation commonly elicited by trauma reminders [3]. The unpredictable occurrence of intrusions and related emotional responses fosters a persistent sense of vulnerability and hyperarousal, even when the memory of the trauma itself has subsided [1]. This symptom cluster disrupts daily functioning, strains interpersonal relationships, and profoundly diminishes quality of life, underscoring the need for effective, targeted interventions [4].

Understanding the real-world dynamics of intrusive memories and their affective concomitants—including the accompanying inner tension—is essential for developing effective interventions. However, much of the current evidence relies on retrospective assessments, which are prone to recall bias and unable to reflect the moment-to-moment variability and contextual sensitivity of these experiences [5,6]. Real-time assessment methods, such as smartphone-based e-diaries, address these limitations by capturing symptoms as they occur in participants’ everyday environments, thereby enhancing ecological validity [7]. Methodological studies have underscored the added value of this approach, showing substantial discrepancies between retrospective and real-time assessments of intrusion frequency [8] and emphasizing the need for real-time, context-sensitive tools, as intrusion rates have been found to vary considerably depending on assessment method [9]. Such fine-grained assessments not only improve the accuracy of symptom monitoring but also provide a sensitive means to evaluate how therapeutic interventions modulate the day-to-day expression of PTSD symptoms, as examined in this study.

Targeting posttraumatic symptoms such as intrusions requires trauma-focused psychotherapies [10], such as dialectical behavior therapy for PTSD (DBT-PTSD) [11,12] or cognitive processing therapy (CPT) [13]. While both treatments have demonstrated efficacy [11,14], they differ in theoretical frameworks and core intervention strategies. DBT-PTSD is a modular, phase-based treatment that integrates trauma-focused cognitive behavioral techniques—most notably structured imaginal exposure—with skills training in emotion regulation and distress tolerance [12]. Within this framework, imaginal exposure serves to deliberately activate trauma memories in a safe and controlled therapeutic context, promoting corrective emotional processing and facilitating habituation to trauma-related cues [11,15]. Prior research has demonstrated that imaginal reliving can reduce both the distress associated with and the frequency of intrusive memories in PTSD [16-18]. The integration of exposure-based and emotion-regulation components in DBT-PTSD is therefore intended to stabilize affective responses while supporting adaptive processing of traumatic experiences.

DBT-PTSD was developed specifically for individuals with complex PTSD presentations and comorbid borderline personality disorder (BPD) features, who often experience severe emotional dysregulation, interpersonal instability, and heightened inner tension in response to stress [11,14]. These emotion-regulation difficulties may amplify the subjective distress elicited by intrusive memories, rendering them particularly destabilizing in everyday life. Patients with co-occurring PTSD and BPD typically exhibit greater overall symptom severity, higher rates of comorbidity, and reduced psychosocial functioning compared with individuals with PTSD alone [19]. Evidence regarding treatment response in this subgroup is mixed: while a meta-analysis found that psychotherapy for PTSD was both feasible and effective in patients with co-occurring BPD [20], other studies have reported higher rates of nonresponse and dropout [21,22]. Overall, while trauma-focused treatments appear beneficial, tailored approaches such as DBT-PTSD may better address the specific challenges associated with PTSD and comorbid BPD symptoms [12,23].

By contrast, CPT targets maladaptive trauma-related beliefs through cognitive restructuring, aiming to alleviate PTSD symptoms by modifying distorted appraisals and corresponding emotions. Building on the parent trial, which found small but statistically significant differences favoring DBT-PTSD over CPT in overall PTSD symptom reduction [14], this analysis investigates whether these between-treatment differences also translate to the everyday experience of specific PTSD symptoms—namely, the frequency of intrusive memories and the intensity of related inner tension—in this clinically complex sample.

This study is based on a multicenter randomized controlled trial (RCT) [14] in which participants completed a 7-day e-diary protocol at 3 time points: pretreatment, after 6 months, and after 12 months of therapy. This analysis includes data from 175 women with PTSD related to childhood sexual or physical abuse, all of whom met at least 3 *Diagnostic and Statistical Manual of Mental Disorders, Fifth Edition (DSM-5)* criteria for BPD in addition to PTSD. Participants were randomized to receive either DBT-PTSD or CPT and completed at least 1 e-diary assessment. Using an event-based design, participants documented intrusive memories and related inner tension in real time, enabling a detailed and ecologically valid picture of symptom trajectories and treatment-related change in this clinically complex population. It was hypothesized that the frequency of intrusive memories and the severity of related inner tension would substantially decrease across treatment in both groups, with more pronounced reductions expected in the DBT-PTSD group compared with the CPT group.

## Methods

### Participants

A total of 175 women diagnosed with PTSD according to *DSM-5* criteria [1] participated in this study. All PTSD diagnoses were specifically linked to experiences of sexual or physical abuse before the age of 18. Participants, aged 18 to 65 years, were recruited as part of a multicenter RCT (German Clinical Trials registration number DRKS00005578), titled “Treating Psychosocial and Neural Consequences of Childhood Interpersonal Violence in Adults.” The trial compared the efficacy of DBT-PTSD and CPT, with detailed treatment protocols published previously [12]. Participants received 1 year of treatment, comprising up to 45 weekly sessions, followed by 3 monthly booster sessions.

Inclusion criteria were a PTSD diagnosis related to childhood abuse and the presence of at least 3 *DSM-5* BPD criteria, including affective instability (criterion 6). Participants were excluded if they had a lifetime diagnosis of schizophrenia or bipolar 1 disorder, intellectual disability, current substance dependence, a BMI below 16.5 kg/m^2^, pregnancy, unstable life circumstances (eg, homelessness), life-threatening suicide attempts within 2 months before enrollment, planned residential treatment, or recent (within the past year) CPT or DBT-PTSD therapy. Participants engaging in high-risk behaviors, continued self-harm, or suicidality (eg, suicide attempts considered to be at low risk of death) were not excluded.

Of the 193 participants initially enrolled in the RCT, 18 (9.3%), including 8 (4.1%) in DBT-PTSD and 10 (5.2%) in CPT, were excluded from the e-diary analysis due to technical problems or incomplete participation. This resulted in a final sample of 175 participants who completed at least 1 e-diary assessment (DBT-PTSD: n=90, CPT: n=85).

### Ethical Considerations

Ethical approval was obtained from the relevant institutional review boards, including the Medical Faculty Mannheim at Heidelberg University (reference 2013‐635 N-MA), as well as the ethics committees of Goethe University and Humboldt University. Written informed consent was obtained from all participants before their inclusion. Safety and data quality were independently monitored by the Coordination Centre for Clinical Trials, Heidelberg. Participants did not receive any compensation.

### Study Procedure

A detailed description of the study protocol and interventions has been published elsewhere [12].

During an initial diagnostic session, trained clinical psychologists assessed inclusion and exclusion criteria, as well as mental health diagnoses. PTSD diagnoses were determined using the Clinician Administered PTSD Scale for *DSM-5* [3]. Comorbid Axis 1 disorders were assessed via the Structured Clinical Interview for *DSM-4* Axis 1 disorders [24], and BPD was evaluated with the International Personality Disorder Examination [25]. Participants also completed self-report questionnaires and provided demographic data, as detailed more extensively for the full sample in the original trial publication [14].

### e-Diary Procedure

At each of the 3 assessment points (baseline, after 6 months, and after 12 months of therapy), participants completed a 7-day e-diary protocol using a study smartphone (Samsung Galaxy S3 mini; Samsung Electronics) equipped with the movisensXS application (movisens GmbH). Before data collection began, participants received standardized instructions and a brief training session to ensure familiarity with the procedure.

The protocol consisted of 2 consecutive phases. During the first phase, which is not part of this manuscript, participants responded for 2 days to time-based prompts that appeared at semirandom intervals, approximately every 30 minutes, between 10 AM and 10 PM. On the evening of the second day (8 PM), participants received an automated reminder message informing them about the transition to the event-based phase starting the following day. This reminder explained that, from day 3 onward, prompts would be reduced to a single morning notification at 9 AM, and participants were instructed to initiate an entry themselves whenever intrusive thoughts or memories of their most distressing traumatic event occurred during the day. The message also reminded participants how to access and activate the event-based entry function (*Thoughts/Memory* button) on the app’s home screen.

During the subsequent 5 days, participants followed the event-based sampling approach. They were instructed to initiate an entry each time they experienced an intrusive memory of their most distressing traumatic event. For the present analyses, only data from the 5-day event-based phase at each assessment point were included.

### e-Diary Assessment

At each self-initiated entry, participants rated their current level of inner tension on a visual analog scale ranging from 0 (*no tension*) to 100 (*maximum tension*) in response to the item: *At this moment, I am experiencing unpleasant inner tension*. They then indicated whether the memory or thought was related to their most distressing traumatic event (*yes* or *no*). Only entries identified as trauma-related were included in the analysis; non-trauma-related entries were automatically discontinued.

All entries were automatically time-stamped, encrypted, and securely transmitted to the study server. The app interface, including the visual analog scale (panel A) and the trauma-relatedness question (panel B), is illustrated in Figure S1 in Multimedia Appendix 1.

### Statistical Analysis

Statistical analyses were conducted in R (R Foundation for Statistical Computing). Baseline levels of intrusion frequency and related inner tension were derived by averaging all available entries from the 5-day pretreatment e-diary period within each participant. Group differences (DBT-PTSD vs CPT) were examined using 2-tailed independent-samples *t* tests.

Subsequent analyses used multilevel modeling to account for the nested data structure (ie, repeated measures within participants). To examine whether treatment group, time, and their interaction predicted the daily frequency of intrusive memories, a multilevel zero-inflated negative binomial model was used. This approach was chosen to appropriately model the characteristics of the outcome variable (intrusion frequency), which exhibited substantial overdispersion and a high proportion of zero values. For momentary tension ratings, a linear mixed-effects model was conducted, with treatment group, time, and their interaction as predictors. A binary dropout variable was included in both models to address potential biases related to missing data.

## Results

### Sample Characteristics

e-Diary data from 175 participants, including 90 (51.4%) in DBT-PTSD and 85 (48.6%) in CPT, were included in the analysis. Across measurement points, participants completed, on average, 11.4 (SD 10.1; range 1‐53) e-diary assessments in the CPT group and 12.0 (SD 9.94; range 1‐50) assessments in the DBT-PTSD group. As presented in Table 1, the treatment groups showed no significant differences in demographic or clinical baseline characteristics.

**Table 1.** Demographic and clinical characteristics at baseline.

	Entire sample (n=175)	DBT-PTSD^a^ (n=90)	CPT^b^ (n=85)	*t* test (*df*)	Chi-square (*df*)	*P* value
Age (y), mean (SD)	35.93 (10.78)	36.81 (10.52)	35.00 (11.03)	–1.11 (170)	—^c^	.27
Education^d^, n (%)				—	1.39 (4)	.85
No graduation or still at school	11 (6.4)	7 (7.8)	4 (4.7)			
Lower secondary school (Hauptschule)	28 (16.3)	16 (17.8)	12 (14.1)			
Intermediate secondary school (Mittlere Reife)	60 (34.9)	29 (32.2)	31 (36.5)			
High school graduation (Abitur)	66 (38.4)	33 (36.7)	33 (38.8)			
Other	7 (4.1)	4 (4.4)	3 (3.5)			
Marital status^e^, n (%)				—	2.36 (2)	.31
Single	83 (48.0)	38 (42.2)	45 (52.9)			
Married or similar relationship	45 (26.0)	23 (25.6)	22 (25.9)			
Separated, divorced, or widowed	45 (26.0)	27 (30.0)	18 (21.2)			
Axis I disorders, mean (SD)						
Current	3.21 (1.44)	3.02 (1.32)	3.41 (1.54)	1.80 (173)	—	.07
Lifetime	4.21 (1.52)	4.06 (1.44)	4.36 (1.60)	1.34 (173)	—	.18
Co-occurring BPD^f^, n (%)	84 (48)	40 (44.4)	44 (51.8)	—	0.67 (1)	.41
BPD criteria, mean (SD)	4.75 (1.57)	4.66 (1.54)	4.85 (1.61)	0.81 (173)	—	.42
Repeated abuse, n (%)	158 (90.3)	78 (86.7)	80 (94.1)	—	1.48 (1)	.22
Age of onset (y), mean (SD)	7.83 (4.19)	7.98 (4.31)	7.67 (4.08)	–0.48 (172)	—	.63
Duration of abuse, mean (SD)	6.76 (5.91)	6.25 (5.29)	7.29 (6.48)	1.17 (172)	—	.24
Perpetrator known to the patient, n (%)	164 (93.7)	86 (95.6)	78 (91.8)	—	0.52 (1)	.47
Psychopathology, mean (SD)						
CAPS-5^g^ total score	40.44 (9.81)	39.70 (10.49)	41.22 (8.90)	1.03 (173)	—	.31
PCL-5^h^ total score	49.03 (10.98)	49.14 (10.79)	48.91 (11.24)	–0.14 (173)	—	.89
Depression severity (BDI-II^i^)	32.97 (10.82)	32.61 (10.93)	33.34 (10.75)	0.44 (173)	—	.66
Level of functioning (GAF^j^)	50.18 (8.55)	50.96 (9.09)	49.35 (7.91)	–1.24 (173)	—	.25
Intrusion frequency per day, mean (SD)	0.99 (1.01)	0.96 (0.97)	1.01 (1.05)	*W*=3919.5^k^	—	.68
Intrusion-related inner tension, mean (SD)	62.48 (23.10)	61.44 (23.84)	63.60 (22.36)	0.61 (170)	—	.54

a DBT-PTSD: dialectical behavior therapy for posttraumatic stress disorder.

b CPT: cognitive processing therapy.

c Not applicable.

d Education data were available for 172 participants.

e Marital status data were available for 173 participants.

f BPD: borderline personality disorder.

g CAPS-5: Clinician-Administered PTSD Scale for *Diagnostic and Statistical Manual of Mental Disorders, Fifth Edition*.

h PCL-5: PTSD Checklist for *Diagnostic and Statistical Manual of Mental Disorders, Fifth Edition*.

i BDI-II: Beck Depression Inventory–II.

j GAF: Global Assessment of Functioning.

k Due to the nonnormal distribution of intrusion frequency data (characterized by a high proportion of zero values and substantial overdispersion), the Mann-Whitney *U* test was used to compare daily intrusion frequencies between the two groups (DBT-PTSD vs CPT).

### Descriptive Statistics

At baseline, participants reported an average of 0.99 (SD 1.01) intrusive memories per day and a mean inner tension score of 62.48 (SD 23.10; scale 0‐100). Baseline levels of intrusive memories and inner tension did not differ significantly between the 2 treatment groups (Table 1).

On average, participants demonstrated reductions in intrusive memory frequency and related inner tension over time, with a more pronounced improvement in inner tension observed in the DBT-PTSD group. At the 12-month assessment, participants in the DBT-PTSD group reported a mean of 0.31 (SD 0.55) intrusions per day and a mean inner tension score of 38.15 (SD 27.56). In comparison, participants in the CPT group reported an average of 0.26 (SD 0.42) intrusions per day and a mean inner tension score of 56.98 (SD 30.51).

### Multilevel Prediction of Intrusion Frequency and Related Inner Tension

For intrusion frequency, a significant main effect of *time* was found, indicating a general reduction in intrusions across both treatment groups (Table 2). However, neither the *group* effect nor the *time* × *group* interaction reached significance. The inclusion of *dropout status* did not significantly alter the results.

**Table 2.** Multilevel models predicting frequency of intrusions per day (model 1) and intrusion-related inner tension (model 2)^a^.

Variable	Estimate	SE	95% CI	β	*P* value
Model 1: frequency of intrusions per day					
Intercept	0.32	0.16	0.02 to 0.63	–0.86	.04
Group	–0.09	0.19	–0.47 to 0.29	0.03	.63
Time	–0.65	0.07	–0.78 to –0.52	–0.53	<.001
Group × time	0.06	0.09	–0.11 to 0.24	0.05	.45
Dropout status	0.15	0.17	–0.19 to 0.48	0.05	.39
Model 2: intrusion-related inner tension					
Intercept	70.93	3.03	65.01 to 76.85	0.003	<.001
Group	4.18	3.81	–3.27 to 11.63	–0.19	.28
Time	–6.11	1.04	–8.15 to –4.07	–0.15	<.001
Group × time	–6.38	1.39	–9.09 to –3.66	–0.16	<.001
Dropout status	–3.26	3.75	–10.59 to 4.07	–0.05	.39

a The number of participants is 175, and the number of observations is 2,025 (model 1) and 2,020 (model 2). Model 1 (frequency of intrusions per day) was analyzed using a multilevel zero-inflated negative binomial regression to account for overdispersion and the high proportion of zero values. For model 2 (intrusion-related inner tension), a linear mixed-effects analysis was conducted.

**Figure 1.**
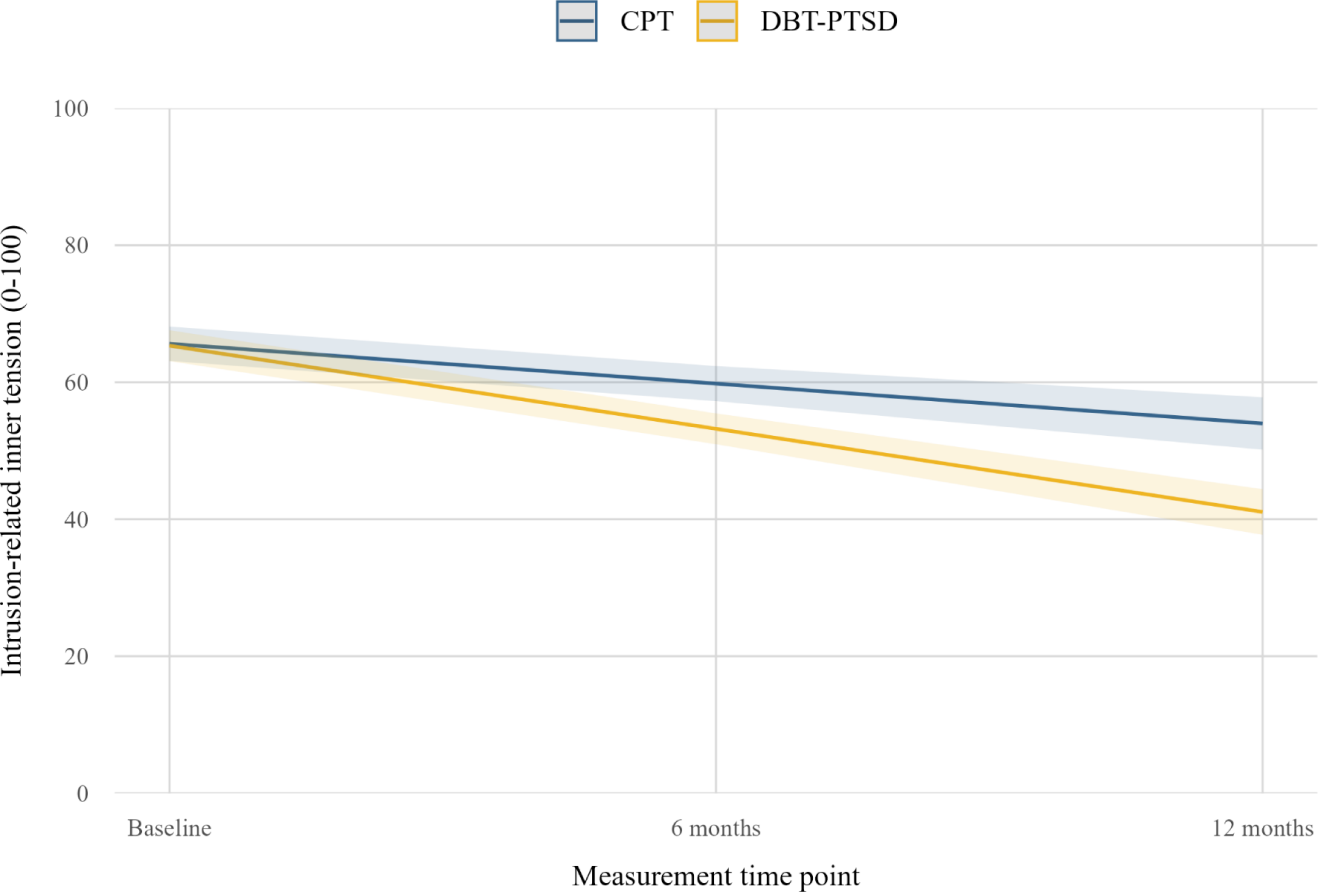
Predicted values for intrusion-related inner tension at 3 time points (baseline, after 6 months, and after 12 months of therapy) by treatment group (dialectical behavior therapy for posttraumatic stress disorder [DBT-PTSD] vs cognitive processing therapy [CPT]). Both groups showed reductions over time, with greater improvements in intrusion-related inner tension observed in the DBT-PTSD group compared to the CPT group.

For intrusion-related inner tension, a significant main effect of *time* also indicated reductions across the study period. Importantly, a significant *time* × *group* interaction was found: participants in the DBT-PTSD group showed significantly greater reductions in inner tension over time compared with those in the CPT group (Table 2 and Figure 1). As with intrusion frequency, neither the main effect of *group* nor *dropout status* were significant. Full parameter estimates from the multilevel models are presented in Table 2.

## Discussion

### Principal Findings

This study used real-time e-diary assessments to evaluate intrusive memories and momentary inner tension experienced during these intrusions in daily life, comparing 2 PTSD treatments—DBT-PTSD and CPT—in a large, clinically complex sample of women with PTSD related to childhood abuse and co-occurring BPD features. By leveraging longitudinal e-diary data, this approach provided a nuanced perspective on individual symptom trajectories while minimizing recall bias and enhancing ecological validity.

Results indicated a significant decrease in intrusive memory frequency over time in both treatment groups, consistent with findings from the parent trial and prior research demonstrating the efficacy of trauma-focused interventions in reducing PTSD symptoms, including intrusions [14,26]. Importantly, the inclusion of participants with co-occurring BPD symptoms underscores the robustness of these improvements in a particularly challenging clinical population. By capturing symptom fluctuations in daily life, this analysis complements the parent trial by illustrating how therapeutic changes manifest in patients’ everyday environments.

While intrusion frequency decreased over time in both treatment groups, no significant group×time interaction emerged. This pattern indicates that both DBT-PTSD and CPT were associated with reductions in daily intrusive memories, without evidence of differential treatment effects. In contrast, the parent trial reported small but statistically significant overall advantages for DBT-PTSD over CPT in clinician-rated PTSD symptom reduction [14]. Notably, the parent trial examined overall symptom severity without analyzing differences at the level of specific symptom clusters, such as intrusions. In addition, this study assessed intrusive memories through self-initiated, momentary reports, whereas the parent trial relied on retrospective clinician ratings encompassing both frequency and intensity. These conceptual and methodological distinctions limit the direct comparability of findings and underscore the value of examining both aggregated and momentary symptom dynamics to better understand treatment-related change. Future research should extend this approach by examining how DBT-PTSD and CPT may differentially influence distinct PTSD symptom clusters in daily life, thereby clarifying how their respective therapeutic mechanisms translate into specific patterns of symptom change.

The most pronounced difference between treatments emerged in the reduction of inner tension reported at the time of intrusive memories, with significantly greater improvements observed in the DBT-PTSD group. This finding may reflect DBT-PTSD’s strong focus on emotion regulation and distress tolerance, which specifically target the heightened arousal and emotional reactivity characteristic of PTSD with comorbid BPD features [1]. Accordingly, these findings extend prior evidence indicating that DBT-PTSD is particularly well suited for this subgroup, while generalization to individuals with PTSD without BPD features should be made with caution. Supporting this interpretation, findings from the parent trial demonstrated greater reductions in dissociation-related tension (Dissociation Tension Scale [27]) and borderline symptoms (Borderline Symptom List 23 [28]) in the DBT-PTSD group compared with the CPT group [14]. Another potential contributor is DBT-PTSD’s use of repeated imaginal exposure, which promotes fear extinction through the activation of trauma-related networks and the integration of corrective experiences [15]. In contrast, CPT primarily targets maladaptive cognitions such as self-blame or distorted worldviews [29], which may be less directly related to immediate reductions in tension. Together, these findings underscore the importance of tailoring interventions to individual symptom profiles, such as the presence of BPD features, to optimize treatment outcomes.

### Limitations

While these findings provide important insights, several limitations should be acknowledged. First, the sample consisted exclusively of cisgender women with PTSD related to childhood abuse and comorbid BPD features, limiting generalizability to other sexes, trauma types, and PTSD without BPD symptoms. Second, although e-diaries allowed for real-time symptom capture, reliance on self-reports may introduce biases. Complementing self-reports with physiological or behavioral measures of arousal or distress could strengthen future research. Third, potential assessment-related reactivity cannot be fully ruled out, although no indication of such effects was observed in the present data. Fourth, the inner tension item may have been interpreted differently across treatments, as “tension” is explicitly addressed in DBT-PTSD but not in CPT, potentially affecting reporting. Fifth, the conceptual relationship between inner tension and general psychological distress requires further empirical investigation to clarify their overlapping and unique aspects. Finally, this study focused on intrusive memories and related inner tension, leaving other PTSD symptoms, such as avoidance and hyperarousal, unexplored. Extending e-diary assessments to these domains could provide a more comprehensive understanding of treatment effects.

### Conclusions

This study is the first to embed e-diary assessments within a large randomized trial of DBT-PTSD and CPT, providing ecologically valid insights into daily-life symptom trajectories in a clinically complex PTSD population with co-occurring BPD features. Both treatments were associated with significant reductions in intrusive memory frequency and momentary inner tension experienced during such episodes, with DBT-PTSD showing greater reductions in the latter. These findings underscore the clinical relevance of both treatments, while suggesting that DBT-PTSD may be particularly beneficial for managing emotional dysregulation and distress in daily life. They further demonstrate that meaningful therapeutic change extends beyond questionnaire-based symptom scores into everyday functioning. Future research should continue to leverage e-diaries to examine how distinct treatment mechanisms translate into real-world recovery across a broader range of PTSD symptoms and in more diverse clinical populations, ultimately helping to refine and individualize treatment approaches for complex trauma presentations.

## Supplementary material

10.2196/81081 Multimedia Appendix 1 Illustration of the e-diary interface and assessment items.

## Abbreviations

BPD: borderline personality disorder
CPT: cognitive processing therapy
DBT-PTSD: dialectical behavior therapy for posttraumatic stress disorder
DSM: Diagnostic and Statistical Manual of Mental Disorders
PTSD: posttraumatic stress disorder
RCT: randomized controlled trial

## References

[1] American Psychiatric Association (2013). Diagnostic and Statistical Manual of Mental Disorders: DSM-5.

[2] Steil R, Ehlers A (2000). Dysfunctional meaning of posttraumatic intrusions in chronic PTSD. Behav Res Ther.

[3] Weathers FW, Bovin MJ, Lee DJ (2018). The Clinician-Administered PTSD Scale for DSM-5 (CAPS-5): development and initial psychometric evaluation in military veterans. Psychol Assess.

[4] Iyadurai L, Visser RM, Lau-Zhu A (2019). Intrusive memories of trauma: a target for research bridging cognitive science and its clinical application. Clin Psychol Rev.

[5] Hackmann A, Ehlers A, Speckens A, Clark DM (2004). Characteristics and content of intrusive memories in PTSD and their changes with treatment. J Trauma Stress.

[6] Michael T, Ehlers A, Halligan SL, Clark DM (2005). Unwanted memories of assault: what intrusion characteristics are associated with PTSD?. Behav Res Ther.

[7] Chun CA (2016). The expression of posttraumatic stress symptoms in daily life: a review of experience sampling methodology and daily diary studies. J Psychopathol Behav Assess.

[8] Priebe K, Kleindienst N, Zimmer J, Koudela S, Ebner-Priemer U, Bohus M (2013). Frequency of intrusions and flashbacks in patients with posttraumatic stress disorder related to childhood sexual abuse: an electronic diary study. Psychol Assess.

[9] Kleindienst N, Priebe K, Petri M (2017). Trauma-related memories in PTSD after interpersonal violence: an ambulatory assessment study. Eur J Psychotraumatol.

[10] Schäfer I, Gast U, Hofmann A, Knaevelsrud C, Lampe A, Liebermann P (2019). S3-Leitlinie Posttraumatische Belastungsstörung.

[11] Bohus M, Dyer AS, Priebe K (2013). Dialectical behaviour therapy for post-traumatic stress disorder after childhood sexual abuse in patients with and without borderline personality disorder: a randomised controlled trial. Psychother Psychosom.

[12] Bohus M, Schmahl C, Fydrich T (2019). A research programme to evaluate DBT-PTSD, a modular treatment approach for complex PTSD after childhood abuse. Bord Personal Disord Emot Dysregul.

[13] Resick PA, Galovski TE, Uhlmansiek MO, Scher CD, Clum GA, Young-Xu Y (2008). A randomized clinical trial to dismantle components of cognitive processing therapy for posttraumatic stress disorder in female victims of interpersonal violence. J Consult Clin Psychol.

[14] Bohus M, Kleindienst N, Hahn C (2020). Dialectical Behavior Therapy for Posttraumatic Stress Disorder (DBT-PTSD) compared with Cognitive Processing Therapy (CPT) in complex presentations of PTSD in women survivors of childhood abuse: a randomized clinical trial. JAMA Psychiatry.

[15] Foa EB, Kozak MJ (1986). Emotional processing of fear: exposure to corrective information. Psychol Bull.

[16] Ehlers A, Clark DM (2000). A cognitive model of posttraumatic stress disorder. Behav Res Ther.

[17] Speckens AEM, Ehlers A, Hackmann A, Clark DM (2006). Changes in intrusive memories associated with imaginal reliving in posttraumatic stress disorder. J Anxiety Disord.

[18] Zoellner LA, Lehinger EA, Rosencrans PL (2023). Brief imaginal exposure for PTSD: trajectories of change in distress. Cogn Behav Pract.

[19] Pagura J, Stein MB, Bolton JM, Cox BJ, Grant B, Sareen J (2010). Comorbidity of borderline personality disorder and posttraumatic stress disorder in the U.S. population. J Psychiatr Res.

[20] Slotema CW, Wilhelmus B, Arends LR, Franken IHA (2020). Psychotherapy for posttraumatic stress disorder in patients with borderline personality disorder: a systematic review and meta-analysis of its efficacy and safety. Eur J Psychotraumatol.

[21] Heffernan K, Cloitre M (2000). A comparison of posttraumatic stress disorder with and without borderline personality disorder among women with a history of childhood sexual abuse: etiological and clinical characteristics. J Nerv Ment Dis.

[22] McDonagh A, Friedman M, McHugo G (2005). Randomized trial of cognitive-behavioral therapy for chronic posttraumatic stress disorder in adult female survivors of childhood sexual abuse. J Consult Clin Psychol.

[23] Kamstra AC, de Vries SO, Brilman MF (2025). Inpatient dialectical behavior therapy combined with trauma-focused therapy for PTSD and borderline personality disorder symptoms: study design of the naturalistic trauma therapy study. Front Psychiatry.

[24] First MB, Williams JB, Karg RS, Spitzer RL (2016). User’s Guide for the SCID-5-CV Structured Clinical Interview for DSM-5 Disorders: Clinical Version.

[25] Loranger AW, Sartorius N, Andreoli A (1994). The international personality disorder examination. The World Health Organization/alcohol, drug abuse, and mental health administration international pilot study of personality disorders. Arch Gen Psychiatry.

[26] Resick PA, Wachen JS, Mintz J (2015). A randomized clinical trial of group cognitive processing therapy compared with group present-centered therapy for PTSD among active duty military personnel. J Consult Clin Psychol.

[27] Stiglmayr C, Schimke P, Wagner T (2010). Development and psychometric characteristics of the Dissociation Tension Scale. J Pers Assess.

[28] Bohus M, Kleindienst N, Limberger MF (2009). The short version of the Borderline Symptom List (BSL-23): development and initial data on psychometric properties. Psychopathology.

[29] Gallagher MW, Resick PA (2012). Mechanisms of change in cognitive processing therapy and prolonged exposure therapy for PTSD: preliminary evidence for the differential effects of hopelessness and habituation. Cognit Ther Res.

