# Supplementary material for: Intrusive Memory Frequency and Related Inner Tension Following Dialectical Behavior Therapy or Cognitive Processing Therapy for Posttraumatic Stress Disorder: An e-Diary Study
Source: JMIR Ment Health. 2025 Dec 8;12:e81081. doi: 10.2196/81081 (PMC12685232; doi:10.2196/81081)
Supplement: Multimedia Appendix 1 [file mental-v12-e81081-s001.docx]

# *Supplemental materials for:*

# Intrusive memory frequency and related inner tension following dialectical behavior therapy or cognitive processing therapy for posttraumatic stress disorder: An e-diary study


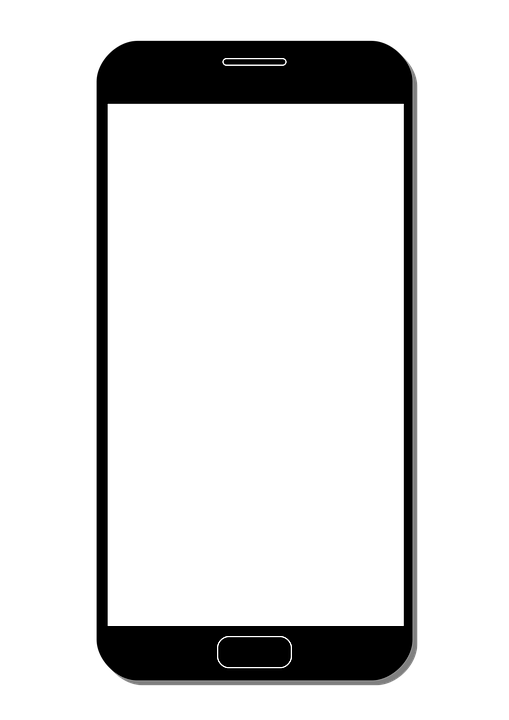


**B**


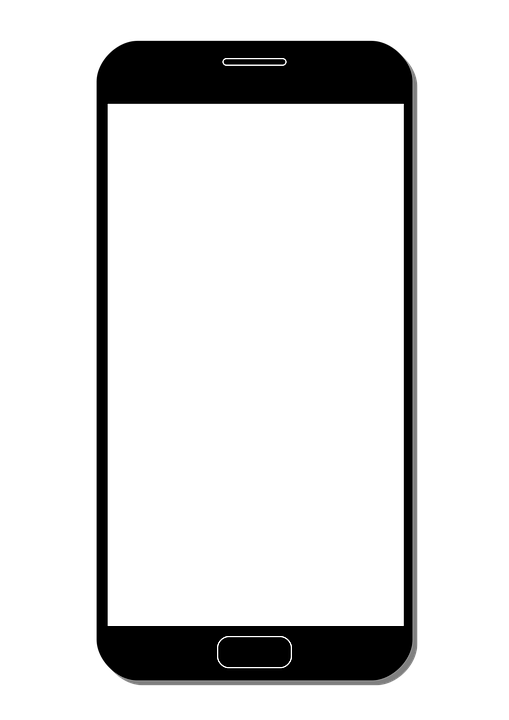


At this moment

- I am experiencing unpleasant inner tension.

**A**

100

0

Did your thoughts or memories relate to your most distressing traumatic experience?

Yes

No

Figure S1. Illustration of the e-diary interface and assessment items.

A) Example of the visual analogue scale used to rate current levels of inner tension ranging from 0 (“no tension”) to 100 (“maximum tension”).

B) Item assessing whether the reported thoughts or memories were related to the participant’s most distressing traumatic experience.
